# Zoonoses in Veterinary Students: A Systematic Review of the Literature

**DOI:** 10.1371/journal.pone.0169534

**Published:** 2017-01-04

**Authors:** Antonio Sánchez, Miranda Prats-van der Ham, Juan Tatay-Dualde, Ana Paterna, Christian de la Fe, Ángel Gómez-Martín, Juan C. Corrales, Antonio Contreras

**Affiliations:** Research Group of Ruminant Health, Animal Health Department, Veterinary School, Regional Campus of International Excellence ‘Campus Mare Nostrum’, Murcia University, Spain; Institut National de la Recherche Agronomique, FRANCE

## Abstract

**Background:**

Veterinary students face diverse potential sources of zoonotic pathogens since the first years of their academic degree. Such sources include different animal species and pathologic materials which are used at university facilities as well as commercial clinics, farms and other external facilities.

**Objectives:**

The present study utilizes a systematic review of the literature to identify zoonoses described in veterinary students.

**Data sources:**

Web of Science and PubMed.

**Results:**

Of the 1,254 titles produced by the bibliographic search, 62 were included in this review. Whereas 28 of these articles (45.2%) described individual cases or outbreaks, the remaining 34 (54.8%) reported serological results. The zoonotic etiological agents described were bacteria, in 39 studies (62.9%), parasites, in 12 works (19.4%), virus, in 9 studies (14.5%) and fungi, in 2 (3.2%) of the selected articles. The selected literature included references from 24 different countries and covered the time period of the last 55 years.

**Limitations:**

The fact that common cases of disease or cases of little clinical importance without collective repercussions are not usually published in peer-reviewed journals limits the possibility to reach conclusions from a quantitative point of view. Furthermore, most of the selected works (66.1%) refer to European or North American countries, and thus, the number of cases due to pathogens which could appear more frequently in non-occidental countries might be underestimated.

**Conclusions/implications:**

The results of the present systematic review highlight the need of including training in zoonotic diseases since the first years of Veterinary Science degrees, especially focusing on biosecurity measures (hygienic measures and the utilization of the personal protective equipment), as a way of protecting students, and on monitoring programs, so as to adequately advise affected students or students suspicious of enduring zoonoses.

## Introduction

Zoonotic diseases are recognized as occupational risks to which veterinarians are subjected [1]. The frequency of veterinarians who acknowledge to have endured a zoonosis varies between 16.7% and 64% [2–4]. Moreover, in these studies the different transmission routes and associated factors occurred in each case are also described. Apart from the communication of clinical cases, the systematic revision of the available scientific literature about zoonoses affecting veterinarians reveals that the seroprevalence against different zoonotic pathogens is greater amongst veterinarians than between the general population, suggesting that veterinarians could act as sentinels to detect emergent diseases and also that they could potentially disseminate zoonotic pathogens to their relatives or the animals they are treating [2].

In general, the activities scheduled in the study plans of Veterinary Sciences are designed with regard to biosecurity guidelines, and additionally, students receive specific advice about the risks associated to animal handling, protective equipment and specific risks associated with pre-existing medical conditions such as immunosuppression or pregnancy [5]. Taking into account the variety of animal species of veterinary interest and the subsequent specialization of veterinary professionals, veterinary students are to face many different sources of zoonotic infections including distinct animal species and in a diversity of situations during their training. In this sense, in addition to the scheduled practices in laboratories, necropsy and dissection rooms, abattoirs and university farms and hospitals, internships in commercial farms, clinics, slaughterhouses or official health services, which are of great academic interest, multiply the chances of interaction between veterinary students and zoonotic pathogens. At the same time, teaching student groups of variable sizes might favor zoonotic outbreaks which in standard conditions would appear as isolated zoonosis cases. Hence, the teaching activity is going to be bound to a changing sanitary situation which will evolve according to the animal species affected and the area of reference. In this context, student’s mobility between educational centers of different regions or countries may expose them to less common pathogens which they are not familiar with, or against which the routinely applied protective measures are less effective.

The vocational character of the veterinary career means that veterinary students have a special relationship with animals, and show great interest in putting knowledge to practical use from the beginning of their education. In this sense, it has been described that the empathy veterinary students have with animals is greater in the first than in the last year of their degree, when they take a more instrumental attitude towards animals [6]. In general, there is a need to update the health risks associated to the teaching activity and also the available protective measures, with special regard to those who belong to any risk group.

The knowledge of zoonotic cases happening in veterinary students dates back a long way. In 1939, Morrill [7] described four cases of infections caused by *Erysipelothrix* spp. in students who got injured during the dissection of a horse. Afterwards, in 1964, Schnurrenberger proposed including the serological detection of different zoonotic pathogens in student’s health programs [8]. Notwithstanding, the global information available about zoonoses in veterinary students is scarce and to date, no systematic review addressing this subject has been published. In addition, the awareness of zoonoses affecting veterinary students could help to design programs aimed at the prevention of this diseases. Therefore, the aim of the present review is to examine and summarize the available scientific literature related to zoonoses in veterinary students.

## Methods

A systematic scientific review, using the PRISMA (Preferred Reporting Items for Systematic Reviews and Meta-Analyses) guidelines [9], of the available literature was performed in September 2016 in order to identify any scientific article documenting zoonotic cases in veterinary students.

### Search Strategy and Selection Criteria

The databases included in the Web of Science^™^ (v5.22.3) and PubMed platforms were consulted. The search in the Web of Science (WOS) included the following databases: Web of Science^™^ Core Collection, Current Contents Connect^®^, KCI-Korean Journal Database, MEDLINE^®^, SciELO Citation Index and Russian Science Citation Index. On both platforms, we used the following Boolean search statements: (zoonoses OR zoonosis OR outbreak OR case OR prevalence OR infection OR antibody) AND (veterinary students).

No time limits were defined and all the articles published in English, French or Spanish with at least an English abstract were selected. Subsequently, the titles and abstracts of the selected articles were examined so as to detect clinical cases or serological evidences of zoonotic diseases in veterinary students. In the articles selected the following data were obtained: zoonosis/causative agent, year (when this information was not specified, the year of publication was considered for guidance), country, number of affected students or percentage of seropositive students, animal species involved, associated risk factors and/or circumstances relevant to each case.

According to the concept of zoonosis, cases of allergies happened during practice lessons, allergies due to vector bites, vaccinal reactions, psychosocial disorders and infections acquired elsewhere unrelated to the academic activity were not considered. Additionally, works of which no English abstracts could be found, although they described zoonoses in veterinary students, were also discarded (these studies were published before 1987).

## Results

**Fig 1:**
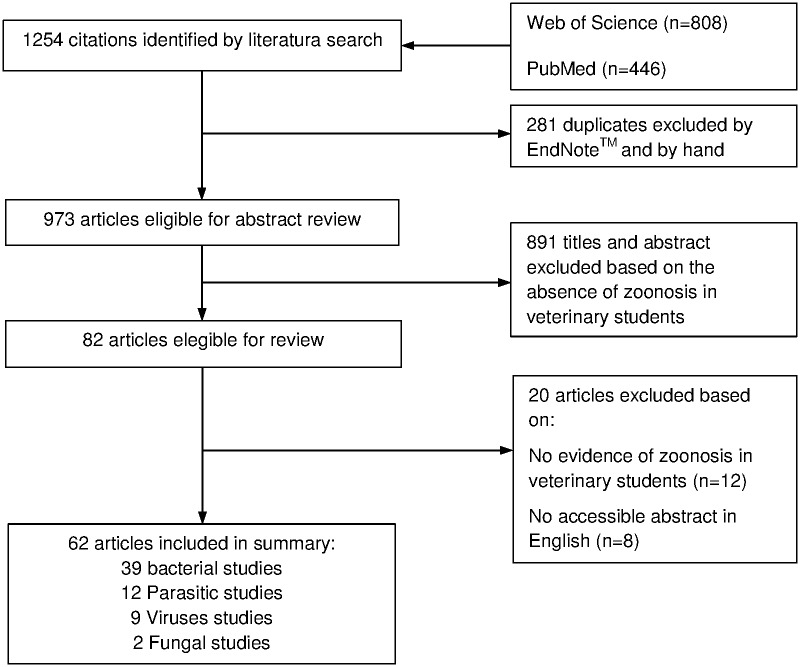
Flowchart of the selection process for publications selected in this review.

This exhaustive revision of the literature provided a total of 1,254 results (808 on WOS and 446 on PubMed), 281 of which were discarded as they were found to be duplicated using a reference manager software (EndNote^™^). These duplications were also confirmed manually. During the review process, 891 works were excluded because they did not confirm disease cases in veterinary students. The remaining 82 studies were analyzed, rejecting those which did not include abstracts in English or which did not provide information according to the established search criteria (Fig 1).

Eventually, a total of 62 articles were selected (Table 1). Fifty-eight of them were written in English, 3 in Spanish and 1 in French, and all of them included an English abstract. Whereas 28 of these articles (45.2%, 95%CI [33.4, 57.5]) described individual cases or outbreaks, the remaining 34 (54.8%, 95%CI [42.5, 66.6]) reported serological results. The zoonotic etiological agents described were bacteria, in 39 studies (62.9%, 95%CI [50.5, 73.8]), parasites, in 12 works (19.4% 95%CI [11.4, 30.9]), virus, in 9 studies (14.5%, 95%CI [7.8, 25.3]) and fungi, in 2 (3.2%, 95%CI [0.9, 11]) of the selected articles. The selected literature included references from 24 different countries and covered the time period of the last 55 years. Twenty-two articles (35.5%, 95%CI [24.7, 47.9]) described zoonoses in veterinary students in Europe, followed by 19 (30.6%, 95%CI [20.6, 43]) which reported cases occurred in the USA, 10 (16.1%, 95%CI [9, 27.2]) in Asia, 7 (11.3%, 95%CI [5.6, 21.5]) in South America and the Caribbean, 3 (4.8%, 95%CI [1.7, 13.3]) in Oceania and 1 case (1.6%, 95%CI [0.3, 8.6]) happened in Africa.

**Table 1:** Zoonoses reported in veterinary students identified by the systematic literature review according to the agent, country, year and main quantitative and qualitative information summarized from the selected studies.

Zoonoses/agent	Country	Year	Human clinical cases or seroprevalence (%)^1^	Animal or risk factors identified and comments	Reference
**Bacteria**					
*Bartonella henselae*	Japan	1995–1999	11.7%	Cat contact has been suggested as a risk factor for cat scratch disease	[10]
*Bartonella henselae*	Japan	1997–1998	2	The clinical cases had association with cat scratch and showed antibody elevation after clinical manifestation of Cat Scratch Disease	[11]
Brucellosis	USA	1959–1964	1, 3.7%	Brucellosis infections were associated to the summer	[8]
Brucellosis	UK	1962–1968	From 8.9% in the first course to 48.5% within five years of graduation	*Brucella abortus* detected by serial *Brucella* agglutination tests	[12]
Brucellosis	France	1968–1982	15.6%-5.2%	Authors discuss the reduction of the seroprevalence in veterinary students in relation with the seroprevalnce of bovine brucellosis in France	[13]
Brucellosis	France	1984	<2% and 5.9%	In veterinary students in three first school years and in their last school year, respectively. % of positive skin test reactions to a phenol-soluble antigen of *Brucella abortus*	[14]
Brucellosis	USA	1984	1	Accidental Inoculation *of Brucella abortus* Strain 19	[15]
Brucellosis	USA	1997	4	The affected students participated in an attempted vaginal delivery, a caesarean delivery, and a necropsy on a stillborn calf that died because of *Brucella abortus* strain RB51 infection	[16]
Brucellosis	India	2005	1.14%	The seroprevalence in veterinary students was lower than in general population (2.45%)	[17]
Brucellosis	Iran	2010	42	Occupational risk was demonstrated	[18]
Brucellosis	Colombia	2010	18.4%	Protective barriers are suggested during contact with animals carrying the organism during training as veterinary medical students	[19]
*Corynebacterium pseudotuberculosis*	USA	1979	1	Affected by pneumonia	[20]
*Corynebacterium pseudotuberculosis*	Norwegian	2007	1	Affected by pneumonia. Laboratory work (possibly due to the inhalation of bacteria when catalase reaction were performed)	[21]
*Corynebacterium ulcerans*	UK	2010	1	Contact with lambing farm or domestic animals were considered to be the most likely sources	[22]
Leptospirosis	USA	1959–1963	0%	Seropositives were not detected for any of the 3 leptospiral serotypes in the 493 serums tested over the 4 year period studied	[8]
Leptospirosis	Spain	1994–1995	8.4% and 11.4% in each period, respectively	Risk factors associated: taking the course specialising in food inspection and technology, on-farm work, contact with pets in general, and particularly carnivores, and contact with animal traders	[23]
Leptospirosis	Colombia	2003	17%	Occupational exposure was identified as a risk factor	[24]
Leptospirosis	Peru	2005	11.9%	Zoonotic origin was suggested	[25]
Leptospirosis	Trinidad and Tobago	2013	9.7%	Veterinary student was the only risk factor that was associated with *Leptospira* infection	[26]
Leptospirosis	New Zealand	2010–2011	0%	Low risk, despite frequent exposure to animal urine	[27]
Lyme disease	Mexico	2016	47.5%	Associated to tick exposure or bites	[28]
Methicillin-resistant *Staphylococcus aureus* (MRSA)	The Netherlands	2006	2 students positives in a population with 3.9% of MRSA carriage	veterinary doctors and students caring for livestock have a high risk of being colonized by MRSA	[29]
Methicillin-resistant *Staphylococcus aureus*	USA	2010	22%	Visiting contaminated pigs farms	[30]
Methicillin-resistant *Staphylococcus aureus*	Malasya	2013	23.3%	Occupational exposure was proposed for MRSA	[31]
*Mycobacterium bovis*	Spain	1986–1990	2	The human cases of tuberculosis by *M*. *bovis* diagnosed in the hospital was the 0.9% of the total of tuberculosis in the period studied	[32]
Psittacosis	USA	1959–1963	0.6%	Psittacosis infection was detected in the spring	[8]
Psittacosis	TheNetherlands	2005	Students infected in a population with an infection frequency of 34%	An outbreak of psittacosis in a veterinary teaching hospital. Parrots, identified as the source of infection, were exposed to a group of cockatiels coming from outside the teaching facility	[33]
Psittacosis	Brazil	2010	1 student seropositive in a population with 23.9% of seropositives	The population studied included veterinarians, biologists, animal scientists, veterinary students, animal keepers and others employees in 20 zoos	[34]
Q fever	USA	1959–1963	5.1%	The higher percentage of seropositives (12.7%) was reached just prior to graduation	[8]
Q Fever	Spain	1994–1995	10.02–11.01%	Coursing the speciality in Food Inspection and technology or the speciality of Animal Production, to practise with living animals (particularly with ruminants) and to contact frequently with persons who worked withanimals	[35]
Q Fever	Turkey	2000	0% in a population with a seroprevalence of 7.8%	Positive results were obtained in farmers, veterinarians and abattoir workers	[36]
Q fever	Slovakia	2011	16.8 and 58% for phase I and II, respectively	Occupational risk factors were suggested	[37]
Q Fever	The Netherlands	2006	18.7%	Study direction “farm animals”, year of study, having had zoonosis, lived on a ruminant farm	[38]
Q fever	The Netherlands	2009	30% of veterinary students in a population of Dutch veterinaries with a seroprevalence of 65.1%	Practical rotations during their study	[39]
Q Fever	Iran	2015	34.7%	Age and sex	[40]
*Streptococcus suis* type 2	New Zealand	1989	0% in a population with a seroprevalence ranged between 9% and 21%	The development of antibody to *S*. *suis* type 2 was associated with occupational contact with pigs or their meat products	[41]
Vancomycin-resistant *Enterococci*	Malasya	2007–2009	4.3%	The populations in close contact with livestock are not at higher risk for the colonization of Vancomycin-resistant Enterococci	[42]
**Parasite**					
*Cryptosporidium* spp.	Finland	1986	5	Associated with contact with experimentally infected calves	[43]
*Cryptosporidium* spp.	USA	1988	10	Associated with direct contact with infected calves and contact with contaminated materials	[44]
*Cryptosporidium* spp.	USA	1987	26	Outbreak in a veterinary teaching hospital after admission of calves from affected farm	[45]
*Cryptosporidium parvum*	USA	1997	2	The index case was an infected dairy calf. Outbreak of cryptosporidiosis occurred at a veterinary hospital involving a pony, a llama and 2 students	[46]
*Cryptosporidium parvum*	USA	2003	7	Calves. Authors recommend considering *Cryptosporidium* spp. as a cause of gastroenteritis among farm-animal workers	[47]
*Cryptosporidium parvum*	New Zealand	2011	25 (attack rate 29%)	Contact with calves during a practical class	[48]
*Cryptosporidium parvum*	UK	2007	6	Associated to a lapse in handwashing procedures on a farm with enzootic *C*. *parvum* in calves	[49]
*Cryptosporidium parvum*	Sweden	2013	13	Entering pens of calves with diarrhoea and eating in clinic cars were identified as risk factors. Washing hands at least twice per farm visit was protective	[50]
*Cryptosporidium parvum*	USA	2015	16	Training session at the bovine obstetric laboratory with euthanized calves	[51]
*Cryptosporidium parvum*	Italy	2013	6	Outbreak associated to two foals hospitalized in an Equine Perinatology Unit	[52]
*Toxocara canis*	France	1988–1989	11.8% and 20.4% in each period, respectively	Hygiene errors and contamination by food were identified as a risk factors	[53]
*Toxocara canis*	Mexico	2008–2010	13%	The seroprevalence in veterinary students were higher than in Graphic Design students in Mexico City (13% and 7.0% respectively)	[54]
*Toxoplasma gondii*	USA	1960–1961	17.8%	The contact with animals and farm environment is discussed as possible risk factors	[55]
*Toxoplasma gondii*	USA	1975–1976	20.4%	No relationships were established between the presence of *T*. *gondii* antibodies and animal contact	[56]
*Toxoplasma gondii*	USA	2002–2006	5.6%	There was no significant difference (P > 0.05) in the prevalence of *T*. *gondii* antibodies in veterinary versus undergraduate students	[57]
*Toxoplasma gondii*	Malasya	2013–2014	14.9%	The age group of ≥ 30 years old and working or study duration of >10 years having close contact with animals were identified as significant risks	[58]
*Toxoplasma gondii*	Iran	2016	33.7%	No statistically significant difference observed in the infection rate between the veterinary laboratory sciences students group and control group	[59]
**Virus**					
Hepatitis E	USA	1999	6% in a population with 23–26% of seropositives	Swine veterinarians (without difference between academic, practicing, student, and industry veterinarians) may be at somewhat higher risk of Hepatitis E virus infection than are normal blood donors	[60]
Poxvirus. Bovine Papular Stomatitis	USA	1979	5	Contacted with cattle. Diagnosed by clinical and epidemiological data	[61]
Poxvirus. Cowpox	Austria	2010	1	The patient had ulcerated nodule in the skin and malaise and a painful pronounced cervical lymphadenopathy	[62]
Poxvirus. Orf	USA	2012	1	Intubation of a goat without wearing gloves	[63]
Poxvirus. Orthopoxvirus	Italy	2005	1	Scratched by a cat	[64]
Rabies	USA	1970–1977	> 200 exposed	Accidental exposure to rabies with an accelerated preexposure rabies prophylaxis program coupled	[65]
Rabies	USA	1979	Students in a group of 36 persons exposed to a rabid dog	Effects of the vaccine types are discussed	[66]
Swine Influenza virus	USA	1981	Students positives in a population with a 11% of seroprevalence	Veterinary students had lower seroprevalence than veterinarians, pork producers and swine abattoir employees	[67]
West Nile Virus	South Africa	2008	1	Transmission during horse autopsy. Handle the brain without mask or eye protection	[68]
**Fungus**					
Dermatophytosis by *Microsporum canis*	Spain	2010	4	Originated in a litter of stray cats	[69]
Dermatophytosis by *Arthroderma vanbreuseghemii*	Switzerland	2015	20	Inadequate immune response of the affected horse and the high number of people in contact with it at the equine clinic were associated with this unusual outbreak	[70]

^1^In veterinary students

The analyzed studies described 21 diseases or infections caused by the same genus or family of pathogenic agents in veterinary students. Cryptosporidiosis was the most frequently described zoonosis (10 articles), followed by brucellosis (9 works), Q fever (7 works), leptospirosis (6 works), toxoplasmosis (5 works), infections by Poxvirus (4 works), methicillin-resistant staphilococcci (3 works), *Corynebacterium* spp. (3 works), *Bartonella henselae* (2 works), dermatophytosis (2 works), psittacosis (2 works), rabies (2 works), *Toxocara canis* (2 works), hepatitis E (1 work), Lyme disease (1 work), *Mycobacterium bovis* (1 work), *Streptococcus suis* type 2 (1 work), Swine Influenza virus (1 work), vancomycin-resistant enterococci (1 work) and West Nile Virus (1 work). The outbreaks with a greater number of diseased students were due to cryptosporidiosis, which caused 26 clinical cases in a veterinary teaching hospital [45]. The accidental exposure to animals infected with rabies virus incurred an intervention on >200 alumni [65]. On the other hand, the greatest seroprevalence values obtained in student groups were reported for Lyme disease (47.5%) [28] and Q fever (30% seropositive students in the Netherlands) [39]. In addition, the serial historic seroprevalence values against brucellosis in veterinary students from the École d’Alfort (France), which describe that the greatest values were obtained in fourth-year students during the school year 1970–71, reaching 37.4% of positive results, is also worth mentioning [13].

All the analyzed articles refer to domestic animals (including stray cats) or their environment, as zoonoses in veterinary students associated to wildlife or exotic animal species have not been reported. Regarding the animal species involved in the cases described in the analyzed literature, calves are held responsible for almost all the cases of cryptosporidiosis [43–51], except one outbreak which was caused by an asymptomatic foal hospitalized in an Equine Perinatology Unit [52]. More specifically, the works about *Bartonella henselae* associate seropositivity against this pathogen or clinical cases of cat-scratch disease with contact or injuries caused by cats, respectively [10, 11]. Porcine livestock has been related to infections such as hepatitis E [60] and swine influencia virus [67], and *Streptococcus suis* Type 2, although this pathogen was not detected in veterinary students which were included in a risk population group [41]. Concerning the animal species associated to infections by Poxviridae virus, ruminants were considered the main source of these agents [61–63], though one student became infected with Orthopoxvirus after being scratched by a cat [64]. Two of the articles that report cases of psittacosis associate this disease to the exposure to birds [33, 34], whereas student’s cases of dermatophytosis were related to contact with cats [69] and an infected horse [70]. In the studies which report the animal species involved in each zoonosis, transmission through direct contact prevails, although indirect infection through contaminated equipment is also described for cryptosporidiosis [50] and dermathopytosis [69].

The occupational nature of zoonotic diseases is clearly discernable considering the works which report cases of brucellosis [8, 12–19], methicillin-resistant staphylococci [29–31] and Q fever [8, 35–40]. Moreover, this relationship is also suggested in various works assessing seropositivity against *Leptospira* spp. in veterinary students [23–26]. One case of infection by *Corynebacterium pseudotuberculosis* of laboratory origin has been reported [21] and also one case of West Nile virus after performing a necropsy on an infected horse [68].

The works reporting infections by *Toxoplasma gondii* [55–59], vancomycin-resistant enterococci [42] and *Toxocara canis* [54] in veterinary students did not demonstrate any association between these infections and the contact with animals.

In the survey performed by de Rooij et al. [38] in the Veterinary Faculty of the University of Utrecht (the Netherlands), students acknowledged to have endured the following zoonotic diseases: Campylobacteriosis (1.5%), Ecthyma (1.3%), Giardiasis (0.1%), Cat scratch (0,4%), Listeriosis (0.3%), Salmonellosis (1.2%), Dermatophytosis (8.5%), other fungal infections (5.5%), *Sthapylococus* (0.7%) and Verotoxigenic *Escherichia coli* (0.3%). However, no cases of Brucellosis, Cryptosporidiosis, Leptospirosis, Psittacosis, Q fever, or Toxoplasmosis were communicated.

## Discussion

The outcomes of the studies analyzed in the present review suggest that the cases of zoonotic diseases in veterinary students are underestimated in the available scientific literature. Most of the 62 selected articles describe outbreaks, clinical cases or serological studies, and only one article provides a survey about self-reported zoonoses in students, which was included in a study of Q fever [38]. This is probably due to the fact that common cases of disease or cases of little clinical importance without collective repercussions are not usually published in peer-reviewed journals, limiting the possibility to reach conclusions from a quantitative point of view. Nevertheless, regarding a qualitative approach, the search criteria applied allowed the identification of a representative number of zoonotic diseases reported in veterinary students. However, the fact that most of the selected works (64%) refer to European or North American countries should be taken into consideration, as the number of cases due to pathogens which appear more frequently in non-occidental countries might be underestimated.

Cryptosporidiosis is the most frequently reported zoonosis, as well as the disease which causes the greatest number of affected students in the associated outbreaks. Generally, the epidemiological characteristics of the cases or outbreaks of cryptosporidiosis are constant between the different studies [43–51], except for one work in which the outbreak was due to two infected foals [52] instead of calves. Insufficient hygienic practices, including inappropriate hand washing (as alcohol-based hand gels are unsuitable), eating inside the cars used to get to the farms and deficient washing temperature of the protective clothing are considered as a risk factor in all these cases [50]. In addition, the fact that oocysts are persistently eliminated in huge amounts to the environment by infected individuals, their great environmental resistance and the relatively low infective dose to human beings (≥10 oocysts) should be taken into account. All these aspects, together with the presence of student groups assisting diseased calves (at commercial or university farms) favor the presentation of outbreaks [43–51]. Not only veterinary students have been affected by cryptosporidiosis; this way, recurrent outbreaks were reported in consecutive semesters causing 31 and 37 cases, respectively, in students between 9^th^– 12^th^ grade from a high school and two middle schools which participated in an educational farm program [71]. In this work, the difficulty of implementing adequate hygienic measures among students is described. Despite the fact that no cases of cryptosporidiosis in veterinary students associated to small ruminants have been reported, preventive advice should also be considered when visiting farms with affected lambs [72].

The results obtained in the different works reporting brucellosis in veterinary students should be temporary and geographically contextualized. The articles relative to France and UK between the 1960s–1980s [12–14] refer to a time period in which animal brucellosis presented high prevalence values and eradication campaigns were being developed. Nowadays, UK is free of bovine and small ruminant brucellosis, whilst in France the whole country is free of bovine brucellosis, 64 departments are officially free of *B*. *melitensis* [73] and no clinical cases of this disease have been reported in small ruminants since 2003. To the contrary, Mediterranean countries still present variable numbers of infected animals and thus, these areas gather the majority of human brucellosis cases in the EU [74]. However, no cases in veterinary students have been reported in these countries in the last years. On the other hand, human cases of brucellosis are currently anecdotic in most of the states of the USA [75]. In contrast, high prevalence values of animal brucellosis are reported in Central America, the Middle East and Asia, which means that veterinary students are at risk in those regions, as pointed out in the articles on this subject [17–19].

The articles reporting Q fever are mainly focused in USA, Europe and Iran. All of them emphasize the occupational risk associated to veterinary students [8, 35–40]. Moreover, these works describe an increase in seroprevalence values when comparing first-year students with students in the last years of their degree. Contact with animals (especially ruminants), contact with people working with animals [35], academic orientation towards large animal specialties, having endured a zoonosis during their degree and having lived at a farm housing ruminants [38]. The Dutch experience clearly demonstrates the link between the impact of Q fever in veterinary students and the situation of the animals regarding this disease. Hence, before the outbreaks in 2007–2009, the mean seroprevalence value in students was 18.7% [38]. This value increased to 30% in 2009 [39]. Additionally, the fact that most of the infections were not notified as they cursed asymptomatically or with mild flulike symptoms must be taken into consideration. Notwithstanding, the negative impact the infection by *Coxiella burnetti* may have, especially on pregnant women and risk groups [76], make it necessary to monitor these collectives precisely. In general, a correct hygiene and utilization of the personal protective equipment (PPE) have been identified as protective factors against infections by *C*. *burnetii* [35]. Nonetheless, those measures may not be sufficient to protect students. After the culling campaign carried out in the Netherlands as a control strategy against Q fever in caprine farms, 17.5% of the workers seroconverted despite their experience using PPE, which promoted the vaccination of this risk group [77]. In this context, serological surveillance of students at risk, and especially those which display compatible symptoms, has been considered [35, 37]. Vaccination with the Australian vaccine is contraindicated in seropositive people [78], although it could be a possibility for seronegative students at the beginning of their degree [39], with special regard to those whose medical history presents a risk of developing chronical forms of Q fever [37]. In any case, doctors looking after veterinary students should take infections by *C*. *burnetii* into consideration in order to realize an early diagnosis and thus avoid the developing of chronical forms of this disease [38, 39].

Fungal infections are the main cause of zoonoses in veterinarians, reaching frequencies which fluctuate between 45.5% [1] and 54.1% [4] of the reported cases of zoonoses. In a survey performed in Canada, 7.6% of the veterinarians declared having endured a mycosis within a 5 year time period [3]. Similarly, dermatophytosis and other fungal infections were the main self-reported zoonoses by veterinary students of the Veterinary Faculty of the University of Utrecht, with a total of 94 cases among the 960 students surveyed [38]. Nevertheless, in the present systematic review only 2 articles reporting dermathomycosis in veterinary students were found, suggesting that the only published cases of fungal infections are those which are etiological, epidemiological or clinical exceptions [69, 70].

The occupational risk associated to veterinary students has also been addressed and reported by works describing infections by methicillin-resistant staphylococci (MRS) [29–31]. Apart from the association of these infections to visiting infected porcine farms [30], the presence of MRS in the clothing worn by hospital personnel in a veterinary teaching hospital has also been demonstrated. Therefore, changing clothes and using disposable equipment when working with carrier animals are recommended [79]. On the contrary, the detection of vancomycin-resistant enterococci in veterinary students has not been associated to contact with livestock but to factors such as age and previous hospitalization [42]. Regarding the works which assess seropositivity against *Leptospira* spp., four articles suggest a zoonotic origin [23–26], whereas only one study states that veterinary students can be defined as a low risk group though their great exposure to animal urine [27] and other of them did not detect any positive over the 4-year period studied [8].

Despite the first evidences associating a greater reactivity of students against toxoplasmas due to their contact with livestock or its environment [55], there is no actual evidence that these veterinary students present a higher risk of infection by *Toxoplasma gondii* than other collectives. Hence, other sources of infection such as food are discussed [55–59]. Likewise, the higher seroprevalence values observed in veterinary students against *Toxocara canis* [54] do not allow to categorically determining the occupational character of those outcomes. Concerning the risk factors associated to this pathogen, errors in hygienic practices and food contamination have been suggested [53].

The consulted literature only described one case of zoonosis in veterinary students originated in a microbiology laboratory [21] and another in a necropsy room [68]. These findings suggest that biosafety protocols are easier to develop in academic facilities than in external farms. The results of the present systematic review highlight the need of including training in zoonotic diseases since the first years of Veterinary Science degrees, or at least before starting with clinical subjects. This training should especially focus on preventive measures, as a way of protecting students. Thus, providing information concerning biosafety regulations and preventive measures is essential to reduce the risk of emergency or re-emergency of zoonoses [80]. In this sense, the Biosecurity Working Group of the Veterinary Faculty of the University of Liège has developed a thorough manual about Biosecurity Standard Operating Procedures, under the direction of Professor Saegerman [81]. This manual describes general biosafety protocols and specific procedures concerning work with different animal species, food science, field practices, experimental farm practices, anatomy department and diagnostic laboratory, including necropsy area and diagnostic imaging. Moreover, all this information is displayed on an adaptive website according to different user profiles (e.g. students, handicapped students, veterinarians, visitors, staff) in the form of an illustrated manual of biosecurity procedures [82]. In the same way, the development and publication of summaries about protective actions, PPE, occupational health and/or control measures against environmental diseases, such as the document recently put to date by the National Association of State Public Health Veterinarians [83], are of special interest. Likewise, campaigns promoting hand hygiene before eating food (based on video displaying, posters and the promotion of hand disinfectants) have proved to be effective between veterinary students, as these campaigns achieved a lasting improvement on their hand hygiene [84]. This training in zoonoses ought to consider special recommendations concerning immunosuppressed students or students with specific medical conditions (e.g. pregnant students). For this, recommendations on the management of animals by immunosuppressed patients [85, 86], may be very useful, as the risk of infection of veterinary students will always be greater than the risk of the owners with the same medical condition. At the same time, Veterinary schools should apply surveillance and monitoring programs so as to advice affected students or students suspicious of enduring zoonoses, allowing the detection and investigation of these cases in order to prevent their further transmission.

## Supporting Information

S1 File PRISMA 2009 checklist. (DOC)

## References

[1] Nienhaus A, Skudlik C, Seidler A. Work-related accidents and occupational diseases in veterinarians and their staff. Int Arch Occ Env Hea. 2005;78: 230–238. 10.1007/s00420-004-0583-5 15776262

[2] Baker WS, Gray GC. Public Veterinary Medicine: Public Health A review of published reports regarding zoonotic pathogen infection in veterinarians. Javma-J Am Vet Med A. 2009;234: 1271–1278. 10.2460/javma.234.10.1271 19442021

[3] Epp T, Waldner C. Occupational health hazards in veterinary medicine: Zoonoses and other biological hazards. Can Vet J. 2012;53: 144–1450. 22851775 PMC3258827

[4] Jackson J, Villarroel A. A Survey of The Risk of Zoonoses for Veterinarians. Zoonoses Public Hlth. 2012;59: 193–201. 10.1111/j.1863-2378.2011.01432.x 21884033

[5] Cockram MS, Aitchison K, Collie DDS, Goodman G, Murray J-A. Animal-handling teaching at the Royal (Dick) School of Veterinary Studies, University of Edinburgh. J Vet Med Educ. 2007;34: 554–560. 10.3138/jvme.34.5.554 18326763

[6] Colombo ES, Pelosi A, Prato-Previde E. Empathy towards animals and belief in animal-human-continuity in Italian veterinary students. Anim Welfare. 2016;25: 275–286.

[7] Morrill CC. Erysipeloid, occurrence among veterinary students. J Infect Dis. 1939;65: 322–324.

[8] Schnurrenberger PR, Helwig JH, Bashe WJ. The incidence of zoonotic infections in veterinary students. J Am Vet Med Assoc. 1964;144: 384–386. 14118025

[9] Moher D, Liberati A, Tetzlaff J, Altman DG, Grp P. Preferred Reporting Items for Systematic Reviews and Meta-Analyses: The PRISMA Statement. Plos Med. 2009;6:6. PMC3090117 21603045

[10] Kikuchi E, Maruyama S, Sakai T, Tanaka S, Yamaguchi F, Hagiwara T, et al Serological investigation of *Bartonella henselae* infections in clinically cat-scratch disease-suspected patients, patients with cardiovascular diseases, and healthy veterinary students in Japan. Microbiol Immunol. 2002;46: 313–316. 12139390 10.1111/j.1348-0421.2002.tb02701.x

[11] Maruyama S, Kabeya H, Nogami S, Sakai H, Suzuki J, Suzuki H, et al Three cases of cat scratch disease diagnosed by indirect immunofluorescence antibody assay and/or polymerase chain reaction of 16S rRNA gene of *Bartonella henselae*. J Vet Med Sci. 2000;62: 1321–1324. 11193351

[12] Cayton HR, Osborne AD, Sylvester DGH. Exposure to *Br abortus* in veterinary undergraduates and graduates. Vet Rec. 1975;97: 447–449. 814683

[13] Pilet C, Person JM. Influence on human brucellosis of the eradication campaign against bovine brucellosis in France—Special case of the students of the Alfort-veterinary-school. B Acad Nat Med Paris. 1983;167: 597–604. 6362796

[14] Duclos PJ, Bentejac MC, Serre A, Bascoul S. Skin-test reactions to a phenol-soluble antigen of Brucella-abortus among veterinary students, Lyon, France, 1984. Int J Epidemiol. 1989;18: 446–450. 2767861 10.1093/ije/18.2.446

[15] Nicoletti P, Ring J, Boysen B, Buczek J. Illness in a veterinary student following accidental inoculation of *Brucella abortus* strain 19. J of Ach. 1986;34: 236–237. 10.1080/07448481.1986.9938944 3086415

[16] Centers for Disease Control and Prevention (CDC). Human exposure to *Brucella abortus* strain RB51-Kansas, 1997. MMWR Morb Mortal Wkly Rep. 1998;47: 172–175. 9518281

[17] Ajay Kumar VJ, Nanu E. Sero-positivity of brucellosis in human beings. Indian J Public Health. 2005;49: 22–24. 15989156

[18] Beheshti S, Rezaian GR, Azad F, Faghiri Z, Taheri F. Seroprevalence of brucellosis and risk factors related to high risk occupational groups in Kazeroon, South of Iran. IJOEM. 2010;1: 62–68. 23022787

[19] Méndez R IA, Trujillo C DM, Duque S CC, Acero M EJ, Cabrera LÁ, Pachón B DP. *Brucella* spp seroprevalence in veterinary medicine students, Bogota, Colombia. Rev Univ Ind Santander Salud. 2013;45: 39–48.

[20] Keslin MH, McCoy EL, McCusker JJ, Lutch JS. *Corynebacterium pseudotuberculosis*—new cause of infectious and eosinophilic pneumonia. Am J Med. 1979;67: 228–231. 463927 10.1016/0002-9343(79)90395-4

[21] Heggelund L, Gaustad P, Havelsrud OE, Blom J, Borgen L, Sundset A, et al *Corynebacterium pseudotuberculosis* pneumonia in a veterinary student infected during laboratory work. Open Forum Infect Dis. 2015;2. 10.1093/ofid/ofv053 PMC4567093 26380345

[22] Taylor J, Saveedra-Campos M, Harwood D, Pritchard G, Raphaely N, Kapadia S, et al Toxigenic *Corynebacterium ulcerans* infection in a veterinary student in London, United Kingdom, May 2010. Eurosurveillance. 2010;15: 8–10. 20738991

[23] Simon MC, Ortega C, Alonso JL, Girones O, Muzquiz JL, Garcia J. Risk factors associated with the seroprevalence of leptospirosis among students at the veterinary school of Zaragoza University. Vet Rec. 1999;144: 287–291. 10204224 10.1136/vr.144.11.287

[24] Gongora A, Parra J, Aponte L, Gomez L. Seroprevalence of *Leptospira* spp in population groups of Villavicencio, Colombia. Rev Salud Publica (Bogota). 2008;10: 269–278. 19039423 10.1590/s0124-00642008000200007

[25] Dammert BN, Noé MN, Falcón PN, Lopera BL, Rodríguez AMdP. Exposure to Leptospira sp. In veterinary students at the beginning and at the end of the career. Rev Investig Vet Peru. 2009;20: 114–119.

[26] James A, Siele K, Harry N, Suepaul S, Stewart-Johnson A, Adesiyun A. Serological evidence of exposure to Leptospira spp. In veterinary students and other university students in Trinidad and Tobago. Interdiscip Perspect Infect Dis. 2013;2013: 719049 10.1155/2013/719049 23365569 PMC3556857

[27] Fang F, Benschop J, Wilson PR, Collins-Emerson JM, Heuer C, Prattley D. Seroprevalence and exposure to risk factors for leptospirosis among veterinary students at Massey University. New Zeal Vet J. 2014;62: 130–135. 10.1080/00480169.2013.862161 24350827

[28] Skinner-Taylor CM, Flores MS, Salinas JA, Arevalo-Nino K, Galan-Wong LJ, Maldonado G, et al Antibody profile to *Borrelia burgdorferi* in veterinarians from Nuevo Leon, Mexico, a non-endemic area of this zoonosis. Reumatologia. 2016;54: 97–102. 10.5114/reum.2016.61208 27504018 PMC4967975

[29] Wulf M, van Nes A, Eikelenboom-Boskamp A, de Vries J, Melchers W, Klaassen C, et al Methicillin-resistant *Staphylococcus aureus* in veterinary doctors and students, the Netherlands. Emerg Infect Dis. 2006;12: 1939–1941. 10.3201/eid1212.060355 17326948 PMC3291345

[30] Frana TS, Beahm AR, Hanson BM, Kinyon JM, Layman LL, Karriker LA, et al Isolation and Characterization of Methicillin-Resistant *Staphylococcus* aureus from Pork Farms and Visiting Veterinary Students. Plos One. 2013;8: e53738 10.1371/journal.pone.0053738 23301102 PMC3536740

[31] Aklilu E, Zunita Z, Hassan L, Cheng CH. Molecular epidemiology of methicillin-resistant *Staphylococcus aureus* (MRSA) among veterinary students and personnel at a veterinary hospital in Malaysia. Vet Microbiol. 2013;164: 352–358. 10.1016/j.vetmic.2013.02.030 23523336

[32] Sauret J, Jolis R, Ausina V, Castro E, Cornudella R. Human tuberculosis due to *Mycobacterium bovis*: report of 10 cases. Tuber Lung Dis. 1992;73: 388–391. 10.1016/0962-8479(92)90046-M 1292721

[33] Heddema ER, van Hannen EJ, Duim B, de Jongh BM, Kaan JA, van Kessel R, et al An outbreak of psittacosis due to *Chlamydophila psittaci* genotype A in a veterinary teaching hospital. J Med Microbiol. 2006;55: 1571–1575. 10.1099/jmm.0.46692-0 17030918

[34] Raso TF, Carrasco AOT, Silva JCR, Marvulo MFV, Pinto AA. Seroprevalence of antibodies to *Chlamydophila psittaci* in zoo workers in brazil. Zoonoses Public Hlth. 2010;57: 411–416. 10.1111/j.1863-2378.2009.01237.x 19538456

[35] Valencia MDS, Rodriguez CO, Punet OG, Giral ID. Q fever seroprevalence and associated risk factors among students from the Veterinary School of Zaragoza, Spain. Eur J Epidemiol. 2000;16: 469–476. 10997835 10.1023/a:1007605414042

[36] Cetinkaya B, Kalender H, Ertas HB, Muz A, Arslan N, Ongor H, et al Seroprevalence of coxiellosis in cattle, sheep and people in the east of Turkey. Vet Rec. 2000;146: 131–136. 10706331 10.1136/vr.146.5.131

[37] Dorko E, Rimarova K, Kecerova A, Pilipcinec E, Dudrikova E, Lovayova V, et al Potential association between *Coxiella burnetii* seroprevalence and selected risk factors among veterinary students in Slovakia. Ann Agr Env Med. 2011;18: 47–53. 21736269

[38] de Rooij MMT, Schimmer B, Versteeg B, Schneeberger P, Berends BR, Heederik D, et al Risk factors of *Coxiella burnetii* (q fever) seropositivity in veterinary medicine students. Plos One. 2012;7: e32108 10.1371/journal.pone.0032108 22363803 PMC3283734

[39] Van den Brom R, Schimmer B, Schneeberger PM, Swart WA, van der Hoek W, Vellema P. Seroepidemiological survey for *Coxiella burnetii* antibodies and associated risk factors in dutch livestock veterinarians. Plos One. 2013;8: e54021 10.1371/journal.pone.0054021 23342063 PMC3546960

[40] Khalili M, Qorbani A, Sharifi H, Golchin M. Prevalence and risk factor of Q fever among veterinary students in Iran. Trop Biomed. 2015;32(4):704–709. 33557462

[41] Robertson ID, Blackmore DK. Occupational exposure to *Streptococcus-suis* type-2. Epidemiol Infect. 1989;103: 157–164. 2776849 10.1017/s0950268800030454 PMC2249482

[42] Getachew Y, Hassan L, Zakaria Z, Zaid CZ, Yardi A, Shukor RA, et al Characterization and risk factors of vancomycin-resistant *Enterococci* (VRE) among animal-affiliated workers in Malaysia. J Appl Microbiol. 2012;113: 1184–1195. 10.1111/j.1365-2672.2012.05406.x 22906187

[43] Pohjola S, Oksanen H, Jokipii L, Jokipii AMM. Outbreak of cryptosporidiosis among veterinary students. Scand J Infect Dis. 1986;18: 173–178. 10.3109/00365548609032325 3704565

[44] Levine JF, Levy MG, Walker RL, Crittenden S. Cryptosporidiosis in veterinary students. J Am Vet Med Assoc. 1988;193: 1413–1414. 3209453

[45] Reif JS, Wimmer L, Smith JA, Dargatz DA, Cheney JM. Human cryptosporidiosis associated with an epizootic in calves. Am J Public Health. 1989;79: 1528–1530. 2817166 10.2105/ajph.79.11.1528 PMC1349807

[46] Konkle DM, Nelson KM, Lunn DP. Nosocomial transmission of Cryptosporidium in a veterinary hospital. J Vet Intern Med. 1997;11: 340–343. 9470158 10.1111/j.1939-1676.1997.tb00477.x

[47] Preiser G. An outbreak of cryptosporidiosis among veterinary science students who work with calves. J Am Coll Health. 2003;51: 213–215. 10.1080/07448480309596353 12822713

[48] Grinberg A, Pomroy WE, Squires RA, Scuffham A, Pita A, Kwan E. Retrospective cohort study of an outbreak of cryptosporidiosis caused by a rare *Cryptosporidium parvum* subgenotype. Epidemiol Infect. 2011;139: 1542–1550. 10.1017/S0950268810002499 21087535

[49] Gait R, Soutar RH, Hanson M, Fraser C, Chalmers R. Outbreak of cryptosporidiosis among veterinary students. Vet Rec. 2008;162: 843–845. 18587060 10.1136/vr.162.26.843

[50] Kinross P, Beser J, Troell K, Silverlas C, Bjoerkman C, Lebbad M, et al *Cryptosporidium parvum* infections in a cohort of veterinary students in Sweden. Epidemiol Infect. 2015;143: 27485–2756. 10.1017/S0950268815000564 PMC4971544 25810126

[51] Drinkard LN, Halbritter A, Nguyen GT, Sertich PL, King M, Bowman S, et al Outbreak of cryptosporidiosis among veterinary medicine students—Philadelphia, Pennsylvania, February 2015. MMWR Morb Mortal Wkly Rep. 2015;64: 773 26203633 10.15585/mmwr.mm6428a7 PMC4584865

[52] Galuppi R, Piva S, Castagnetti C, Sarli G, Iacono E, Fioravanti ML, et al *Cryptosporidium parvum*: From foal to veterinary students. Vet Parasitol. 2016;219: 53–56. 10.1016/j.vetpar.2016.02.001 26921039

[53] Baixench MT, Magnaval JF, Dorchies P. Epidemiology of toxocariasis in students of the national-veterinary-school in Toulouse. Rev Med Vet-Toulouse. 1992;143: 749–752.

[54] Heredia R, Romero C, Mendoza GE, Ponce M, Portal A, Gonzalez L, et al Ocurrence of *Toxocara canis* in students of veterinary and graphic design in a mexican university. Acta Scientiae Veterinariae. 2014;42: 1219

[55] McCulloch WF, Braun JL, Heggen DW, Top FH. Studies on medical and veterinary students skin tested for toxoplasmosis. Public Health Rep. 1963;78: 689–698. 14049189 PMC1915300

[56] Zimmermann WJ. Prevalence of *Toxoplasma-gondii* antibodies among veterinary college staff and students, iowa-state-university. Public Health Rep. 1976;91: 526–532. 825918 PMC1440572

[57] Rosypal AC, Houk AE, Zajac AM, Lindsay DS. Prevalence of IgG antibodies to *Toxoplasma gondii* in veterinary and undergraduate students at virginia tech, Blacksburg, Virginia. Zoonoses Public Hlth. 2015;62: 553–556. 10.1111/zph.12184 25753511

[58] Brandon-Mong G-J, Seri NAACM, Sharma RS-K, Andiappan H, Tan T-C, Lim YA-L, et al Seroepidemiology of toxoplasmosis among people having close contact with animals. Front.Immunol. 2015;6: 143 10.3389/fimmu.2015.00143 25972863 PMC4412133

[59] Sadaghian M, Jafari R. Prevalence of toxoplasma infection in veterinary laboratory sciences students comparing to ordinary people: a case-control study. J Parasit Dis. 2016;40: 768–771. 10.1007/s12639-014-0575-7 27605781 PMC4996188

[60] Meng XJ, Wiseman B, Elvinger F, Guenette DK, Toth TE, Engle RE, et al Prevalence of antibodies to hepatitis E virus in veterinarians working with swine and in normal blood donors in the United States and other countries. J Clin Microbiol. 2002;40: 117–122. 10.1128/JCM.40.1.117-122.2002 11773103 PMC120098

[61] Schnurrenberger PR, Swango LJ, Bowman GM, Luttgen PJ. Bovine papular stomatitis incidence in veterinary students. Can J Comp Med. 1980;44: 239–243. 6253033 PMC1320068

[62] Glatz M, Richter S, Ginter-Hanselmayer G, Aberer W, Muellegger RR. Human cowpox in a veterinary student. Lancet Infect Dis. 2010;10: 288 10.1016/S1473-3099(10)70054-2 20334852

[63] Simmons JF, Hafernick AC. Painless, red nodule on the finger of a veterinary student. Am Fam Physician. 2012;86: 77–79. 22962916

[64] Carletti F, Bordi L, Castilletti C, Di Caro A, Falasca L, Gioia C, et al Cat-to-Human Orthopoxvirus Transmission, Northeastern Italy. Emerg Infect Dis. 2009;15: 499–500. 10.3201/eid1503.080813 19239778 PMC2681114

[65] Russell LH, Goswick CB, Flowers AI. Accidental exposure of veterinary students to rabies. J Am Vet Med Assoc. 1977;171: 1184–1186. 924837

[66] Dempster G, Stead S, Zbitnew A, Rhodes AJ, Zalan E. Management of 41 persons exposed to a rabid dog: unplanned experience with human diploid vaccine. Can Med Assoc J. 1979;120: 1069–1074. 445300 PMC1819267

[67] Woods GT, Schnurrenberger PR, Martin RJ, Tompkins WAF. Swine influenza-virus in swine and man in Illinois. J Occup Environ Med. 1981;23: 263–267.68. 6260919

[68] Venter M, Steyl J, Human S, Weyer J, Zaayman D, Blumberg L, et al Transmission of West Nile virus during horse autopsy. Emerg Infect Dis. 2010;16: 573–575. 10.3201/eid1603.091042 20202454 PMC3322023

[69] Hermoso-de-Mendoza M, Hermoso-de-Mendoza J, Alonso JM, Rey JM, Sanchez S, Martin R, et al A zoonotic ringworm outbreak caused by a dysgonic strain of *Microsporum canis* from stray cats. Rev Iberoam Micol. 2010;27: 62–65. 10.1016/j.riam.2009.12.007 20346301

[70] Chollet A, Wespi B, Roosje P, Unger L, Venner M, Goepfert C, et al An outbreak of *Arthroderma vanbreuseghemii* dermatophytosis at a veterinary school associated with an infected horse. Mycoses. 2015;58: 233–238. 10.1111/myc.12301 25676308

[71] Kiang KM, Scheftel JM, Leano FT, Taylor CM, Belle-Isle PA, Cebelinski EA, et al Recurrent outbreaks of cryptosporidiosis associated with calves among students at an educational farm programme, Minnesota. Epidemiol Infect. 2006;134: 878–886. 10.1017/S0950268805005649 16672084 PMC2870455

[72] Utsi L, Smith SJ, Chalmers RM, Padfield S. Cryptosporidiosis outbreak in visitors of a UK industry-compliant petting farm caused by a rare *Cryptosporidium parvum* subtype: a case-control study. Epidemiol Infect. 2016;144: 1000–1009. 10.1017/S0950268815002319 26424385

[73] Commision Decision of 21 December 1992 recording the compliance by certain Member States or regions with the requirements relating to brucellosis (B. melitensis) and according them the status of a Member State or region officially free of the disease. OJ L 13, p. 14.

[74] EFSA (European Food Safety Authority) and ECDC (European Centre for Disease Prevention and Control). The European Union summary report on trends and sources of zoonoses, zoonotic agents and food-borne outbreaks in 2014. EFSA Journal. 2015;13: 191.

[75] Centers for Disease Control and Prevention (CDC). Brucellosis Atlanta, GA: U.S. Department of Health and Human Services, https://www.cdc.gov/brucellosis/ [September, 2016].

[76] Million M, Raoult D. Recent advances in the study of Q fever epidemiology, diagnosis and management. J Infection. 2015;71:S2–S9. 10.1016/j.jinf.2015.04.024 25917809

[77] Whelan J, Schimmer B, Schneeberger P, Meekelenkamp J, Ijff A, van der Hoek W, et al Q Fever among culling workers, the Netherlands, 2009–2010. Emerg Infect Dis. 2011;17: 1719–1723. 10.3201/eid1709.110051 21888803 PMC3322078

[78] Gidding HF, Wallace C, Lawrence GL, McIntyre PB. Australia's national Q fever vaccination program. Vaccine. 2009;27:2037–2041. 10.1016/j.vaccine.2009.02.007 19428827

[79] Singh A, Walker M, Rousseau J, Monteith GJ, Weese JS. Methicillin-resistant staphylococcal contamination of clothing worn by personnel in a veterinary teaching hospital. Vet Surg. 2013;42: 643–648. 10.1111/j.1532-950X.2013.12024.x 23662728

[80] Saegerman C, Dal Pozzo F, Humblet M. Reducing hazards for humans from animals: emerging and re-emerging zoonoses. IJPH. 2012;9:13–24.

[81] Biosecurity Working Group. Biosecurity Standard Operating Procedures (SOP) applied to the Faculty of Veterinary Medicine of the University of Liège (FVM) http://www2.fmv.ulg.ac.be/actualites/Biosecurity_Manual_Final_6Jan10.pdf10 [December, 2016].

[82] University of Liege's Faculty of Veterinary Medicine Biosecurity Unit and the Multimedia Workshop. Biosecurity SOP for the ULg Faculty of Veterinary Medicine: http://www.fmv-biosecurite.ulg.ac.be/index.php; [December, 2016].

[83] Williams CJ, Scheftel JM, Elchos BL, Hopkins SG, Levine JF. Compendium of veterinary standard precautions for zoonotic disease prevention in veterinary personnel. Javma-J Am Vet Med A. 2015;247: 1252–1277. 10.2460/javma.247.11.1252 26594810

[84] Heinrich ER, KuKanich KS, Davis E, White BJ. Public health campaign to promote hand hygiene before meals in a college of veterinary medicine. J Vet Med Educ. 2014;41: 301–310. 10.3138/jvme.0913-124R1 24981423

[85] Hemsworth S, Pizer B. Pet ownership in immunocompromised children-A review of the literature and survey of existing guidelines. Eur J Oncol Nurs. 2006;10: 117–127. 10.1016/j.ejon.2005.08.001 16581294

[86] Pena A, Abarca K, Weitzel T, Gallegos J, Cerda J, Garcia P, et al One Health in Practice: A pilot project for integrated care of zoonotic infections in immunocompromised children and their pets in Chile. Zoonoses Public Hlth. 2016;63: 403–409. 10.1111/zph.12241 26684576

